# Longitudinal Prospective Association between Hedonic Hunger and Unhealthy Food and Drink Intake in Adolescents

**DOI:** 10.3390/ijerph17249375

**Published:** 2020-12-15

**Authors:** Tyler B. Mason, Kathryn E. Smith, Jason M. Lavender, Adam M. Leventhal

**Affiliations:** 1 Department of Preventive Medicine, University of Southern California, Los Angeles, CA 90032, USA; adam.leventhal@usc.edu; 2 USC Institute for Addiction Science, University of Southern California, Los Angeles, CA 90032, USA; ksmith41@usc.edu; 3 Department of Psychiatry and Behavioral Sciences, University of Southern California, Los Angeles, CA 90033, USA; 4 Department of Medicine, Uniformed Services University of the Health Sciences, Bethesda, MD 20814, USA; jason.lavender.ctr@usuhs.edu; 5 Military Cardiovascular Outcomes Research (MiCOR) Program, Bethesda, MD 20814, USA; 6 Metis Foundation, San Antonio, TX 78205, USA

**Keywords:** hedonic hunger, eating, unhealthy eating, eating behavior, food intake, drink, adolescents, youth

## Abstract

Hedonic hunger (i.e., extreme responsiveness to food) has been associated with obesity and poor diet, but findings in the existing literature have primarily been cross-sectional. The current study examined hedonic hunger as a prospective predictor of the longitudinal trajectory of unhealthy food and drink intake across adolescence. Ninth grade students from Los Angeles area high schools (analyzed sample *N* = 3268) completed paper-and-pencil surveys across four annual waves; hedonic hunger was assessed at Wave 1, and unhealthy food and drink intake was assessed at Waves 1–4. Multilevel models showed a significant main effect of hedonic hunger and interactions between hedonic hunger and the linear and quadratic components of time. The association between hedonic hunger and unhealthy food and drink intake was strongest at Wave 1 and weakened over time, yet those with higher hedonic hunger reported greater unhealthy food and drink intake across the four years. Efforts to prevent and intervene upon hedonic hunger and its association with unhealthy food and drink intake during childhood and adolescence are warranted.

## 1. Introduction

Unhealthy food and drink intake, particularly the intake of processed foods rich in added sugars and saturated fat, and fast food, is highly prevalent among adolescents globally [1,2,3]. In addition, marketing of unhealthy foods and drinks to adolescents through social media and other methods is ubiquitous [4]. High intake of unhealthy foods and drinks is associated with lower overall dietary quality, increased weight gain, and greater chronic disease risk [5,6,7]. 

Importantly, adolescence represents a key developmental stage for investigating emerging patterns of unhealthy food and drink intake as this is a period during which youth become increasingly autonomous in their eating-related decisions [8,9]. Along with experiencing increased autonomy around food and drink intake more generally, adolescents also have greater autonomy specifically with regard to eating-related decisions outside their own home food environment [8,9]. For instance, adolescents may go to food and beverage outlets near their home or school without parental supervision, and, as adolescents reach driving age, their access to a larger number of restaurants and fast food outlets in and near their community increases [10,11,12]. Consistently, research has shown a high degree of fast food intake among adolescents [4]. Understanding trait-based factors that concurrently and prospectively predict unhealthy food and drink intake among adolescents has the potential to increase our knowledge of adolescents’ risk for the development of poor diet, which is key for prevention of future excess weight gain and obesity-related chronic diseases [13].

One salient trait-based factor is hedonic hunger—characterized by excessive responsivity to, pleasure toward, and drive for food, typically in the absence of physiological hunger [14]. Much of the research on hedonic hunger has been conducted in adults, and studies have shown positive associations between hedonic hunger and loss of control eating, but inconsistent associations with measures of food intake [15]. There have been several studies on hedonic hunger and food intake in adolescents. Cross-sectional data in adolescents aged 10–17 years demonstrated a positive association between hedonic hunger and unhealthy snacking [16], and ecological momentary assessment data among adolescents aged 13–18 years showed positive associations between hedonic hunger and unhealthy food intake [17]. While these studies provide preliminary evidence supporting the potential role of hedonic hunger in relation to unhealthy food intake in youth, research has yet to examine the extent to which hedonic hunger prospectively predicts the trajectory of unhealthy food and drink intake across adolescence. 

Given the novel food and drink options available to adolescents and the increasing autonomy across this developmental period, adolescent youth higher in hedonic hunger—who receive especially high pleasure and reward from food—may be more likely to consume more unhealthy food and drinks compared to youth who are lower in hedonic hunger. Further, hedonic hunger may predict greater increases in unhealthy food and drink intake over the course of adolescence, which may be due to adolescents increasingly being able to access novel food and drink outlets as they get older. Increased access to a greater number of food and drink outlets across adolescence likely emerges along with developmental changes in independence that are common during this period, including learning to drive on one’s own, being able to stay out later, and parents allowing adolescents greater choice in the places they can go on their own. However, whether hedonic hunger increases unhealthy food and drink longitudinally has yet to be explored empirically.

In order to examine baseline hedonic hunger as a prospective predictor of adolescents’ unhealthy food and drink intake, the current paper used data from a large cohort study of adolescents who completed measures in ninth grade (baseline) and then annually for three years of follow-up (grades 10–12). Baseline hedonic hunger was examined as a predictor of trajectory of unhealthy food and drink intake across the high school years. It was hypothesized that higher levels of hedonic hunger at baseline would predict elevated unhealthy food and drink intake longitudinally. Given that developmental trajectories are often non-linear [18,19,20], interactions between baseline hedonic hunger and the linear and quadratic components of wave were explored.

## 2. Method

### 2.1. Participants and Procedures

Participants were ninth grade high school students recruited from ten Los Angeles area high schools in 2013. Students were part of the Health & Happiness cohort study, which focused on studying mental and physical health and health behaviors in adolescents. Recruitment was completed using a convenience sampling approach; more details about this approach have been described elsewhere [21]. Students completed paper-and-pencil surveys in their school classroom. Those who were not in attendance during data collection completed abbreviated surveys by telephone, Internet, or mail; unhealthy food and drink intake were omitted from these measures at Waves 2, 3, and 4. The study protocol was reviewed and approved by the University of Southern California Institutional Review Board (#HS-12–00180). Adolescent participants provided their assent to participate, and their parents provided informed consent for their child to participate in the study.

The current study used data from baseline (Wave 1; fall 9th grade, 2013; *N* surveyed = 3396), 12-month follow-up (Wave 2; fall 10th grade, 2014; *N* surveyed = 3281; 3.4% not surveyed), 24-month follow-up (Wave 3; fall 11th grade, 2015; *N* surveyed = 3232; 4.8% not surveyed), and 36-month follow-up (Wave 4; fall 12th grade, 2016; *N* surveyed = 3168; 6.8% not surveyed). 

### 2.2. Measures

*Demographics.* Adolescents self-reported gender, age, race, height, weight, and mother and father educational attainment. Body mass index (BMI) was calculated with self-reported height and weight using the standard formula (kg/m^2^). Adolescents reported on the education level for each parent, and the highest parental education from either parent was used to represent highest parental education. 

*Baseline hedonic hunger.* The Children’s Power of Food Scale (C-PFS) [22] was used to measure hedonic hunger at Wave 1. Adolescents rated 11 items on a Likert scale ranging from 1 (strongly disagree) to 5 (strongly agree). Sample C-PFS items were: “I feel like food controls me instead of me controlling my food choices” and “Just before I taste a favorite food, I get very excited”. Items were averaged to calculate the overall score, and higher scores reflected greater trait-level hedonic hunger at baseline. The C-PFS has demonstrated adequate psychometric properties among youth [22], and Cronbach’s alpha in the current study was 0.95, which suggests appropriate internal consistency.

*Unhealthy food and drink intake.* The National Cancer Institute’s (NCI) Quick Food Scan was used to measure unhealthy food and drink intake at Waves 1–4 [23]. This adapted version was comprised of food and drink items from the School Physical Activity and Nutrition Survey [24]. Food and drink items included sweet foods (3 items; i.e., candy, frozen desserts, and baked goods/pastries), high fat foods (6 items; i.e., red and processed meat, fried foods, tacos/burritos/enchiladas, cheese, pizza, and French fries/chips), and sweet drinks (2 items; i.e., soft drinks/diet soda and fruit-flavored drinks/sports drinks). Participants indicated how frequently they consumed each food and drink item over the past 12 months on a scale ranging from 0 (never) to 7 (2 or more times per day). 

The NCI guidelines were used to transform each item into a daily consumption score by standardizing the midpoint of each frequency category to the number of times per day. For each of the three food categories, a maximum daily consumption score was computed and served as the indicator of dietary intake for that the respective food category. For example, if an adolescent reported consuming soft drinks/diet soda “2 times a day” but reported not drinking any other sweet drinks, “2 times a day” was used as the value for sweet drinks. The three food categories were then averaged to form a mean representing a total unhealthy food and drink score, with higher scores indicating more unhealthy food and drink intake. This measure has shown adequate validity when compared to 24-h dietary recall [25].

### 2.3. Statistical Analyses

Analyses were conducted in SPSS version 25 (IBM; Armonk, NY, USA). Descriptive statistics were first calculated. A two-level multilevel model (i.e., adolescents nested within wave), accounting for non-independence of observations in the data [26], was used to analyze the data. Multiple imputation [27] was implemented to impute data for missing demographic covariates, and pooled estimates from five imputation datasets were reported. Multilevel models examined the main effects of baseline hedonic hunger and wave and two-way interactions between baseline hedonic hunger and the linear and quadratic wave terms as predictors of unhealthy food and drink intake. Inclusion of the quadratic wave and baseline hedonic hunger × quadratic wave interaction terms allowed for exploring non-linear associations [20]. Specifically, these terms modeled the rate of acceleration or deceleration of unhealthy food and drink intake across time and how this differs across levels of baseline hedonic hunger. Analyses controlled for baseline demographic variables including gender, age, race, BMI, and highest parental education. Alpha was set at *p* < 0.05. Supplementary analyses were run with these models for each of the individual unhealthy food and drink subscales (i.e., sweet foods, high fat foods, and sweet drinks).

## 3. Results

Of the original 3396 participants who completed surveys at baseline (Wave 1), 127 participants were removed due to missing data for baseline hedonic hunger, and one participant was removed due to missing data for unhealthy food and drink intake at all waves. The final sample was thus comprised of 3268 participants. Of these, 3263 participants had unhealthy food and drink intake data at Wave 1, 2835 at Wave 2, 2574 at Wave 3, and 2450 at Wave 4. Missing data for unhealthy food and drink intake at each wave were a result of factors including non-attendance at school or skipping the unhealthy food and drink measurements.

At Wave 1, the mean age of the sample was 14.08 years (*SD* = 0.41; Range: 12–16). A total of 46.6% of the adolescents were boys, and 53.4% were girls. The mean BMI was 21.66 kg/m^2^ (*SD* = 4.33; Range: 12.22–47.87). The racial/ethnic breakdown of the sample was 48.3% Hispanic, 16.4% White, 16.8% Asian, 6.8% Multiracial, 5.0% Black or African-American, 4.2% Native Hawaiian or Pacific Islander, 1.6% Other, and 1.0% American Indian or Alaskan Native. For highest parental education, 13.7% had less than eighth grade, 3.4% had some high school, 7.8% had a high school degree, 14.5% had some college, 16.9% had a college degree, 27.3% had an advance degree, and 16.3% were unknown. The mean hedonic hunger score at Wave 1 was 2.39 (*SD* = 0.96; Range: 1–5). Table 1 displays descriptive statistics and bivariate correlations among study variables. 

Table 2 displays results from the multilevel model of baseline hedonic predicting trajectory of unhealthy food and drink intake in adolescents. With regard to covariates, boys had lower unhealthy food and drink intake compared to girls (*B* = −0.12, *SE* = 0.02, *p* < 0.001), and adolescents whose parent(s) had a higher level of education also demonstrated lower unhealthy food and drink intake (*B* = −0.02, *SE* = 0.01, *p* = 0.004). Compared to non-Hispanic White adolescents, Asian adolescents had lower unhealthy food and drink intake (*B* = −0.30, *SE* = 0.04, *p* < 0.001), and Black adolescents had higher unhealthy food and drink intake (*B* = 0.22, *SE* = 0.05, *p* < 0.001). Older adolescents had lower unhealthy food and drink intake (*B* = −0.05, *SE* = 0.3, *p* = 0.03). BMI was not significantly associated with unhealthy food and drink intake. 

**Figure 1:**
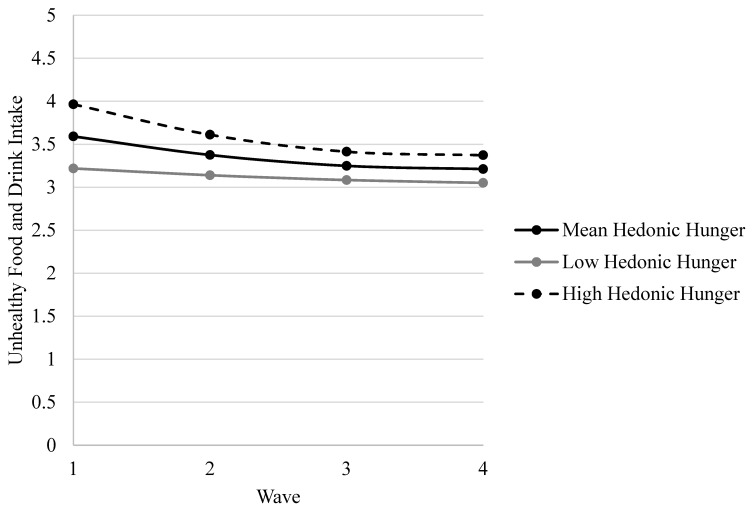
Mean unhealthy food and drink intake score trajectories across time, by baseline hedonic hunger level.

There was a significant main effect of baseline hedonic hunger (*B* = 0.60, *SE* = 0.05, *p* < 0.001), such that greater baseline hedonic hunger scores were associated with more unhealthy food and drink intake in aggregate. There were no significant main effects for the linear effect of wave (*B* = 0.24, *SE* = 0.13, *p* = 0.05) nor the quadratic effect of wave (*B* = −0.04, *SE* = 0.03, *p* = 0.13). Significant interactions were found between baseline hedonic hunger and the linear component of wave (*B* = −0.25, *SE* = 0.05, *p* < 0.001) and the quadratic component of wave (*B* = 0.04, *SE* = 0.01, *p* < 0.001). Figure 1 depicts the interaction of baseline hedonic hunger and wave in relation to unhealthy food and drink intake. The positive association between baseline hedonic hunger and unhealthy food and drink intake was strongest at baseline, which is evident in the figure based on the larger mean differences in unhealthy food and drink intake across low, mean, and high baseline hedonic hunger scores at Wave 1 versus at subsequent waves; this is also evident in the bivariate correlations between baseline hedonic hunger and unhealthy food and drink intake across waves reported in Table 1. 

The linear wave × baseline hedonic hunger interaction showed that the association between baseline hedonic hunger and unhealthy food and drink intake weakened over time, and the quadratic wave × baseline hedonic hunger interaction showed that adolescents with high baseline hedonic hunger demonstrated a greater deceleration in this association. Yet greater baseline hedonic hunger was consistently associated with more unhealthy food and drink intake across the four years. Specifically, those high in hedonic hunger at baseline showed decreasing unhealthy intake that later stabilized over time, whereas those low in hedonic hunger at baseline showed relative stability in unhealthy intake across waves. Supplementary Tables S1–S3 showed that the primary results for baseline hedonic hunger and interactions between baseline hedonic hunger and the linear and quadratic components of wave were consistent across unhealthy food and drink subscales; only the significance of certain covariates differed across models.

## 4. Discussion

In this study, we aimed to examine how baseline hedonic hunger predicted unhealthy food and drink concurrently and longitudinally across adolescence. The findings from this investigation were consistent with the hypothesis that baseline hedonic hunger would be related to more unhealthy food and drink intake and showed that hedonic hunger is a relevant trait-based factor associated with patterns of unhealthy food and drink intake across the adolescent years. The association was strongest concurrently at baseline, demonstrated by larger differences in unhealthy food and drink intake means between those with low, average, and high baseline hedonic scores at Wave 1, and weakened across the four years; however, adolescents with higher baseline hedonic hunger continued to report more unhealthy food and drink intake across all waves. These results suggest that adolescents who are especially responsive to and driven by food are more prone to consuming unhealthy foods and drinks that are typically highly palatable and calorically dense, and this tendency appears to be at least somewhat persistent over time. Further, it is possible that hedonic hunger may over time increase liking for these unhealthy foods and drinks [28] and decrease ability to control consumption [29].

The association between baseline hedonic hunger and unhealthy food and drink was strongest at Wave 1, consistent with the fact that both were measured at this wave. There are several possible explanations for the finding that the association weakened over time. First, it may be reflective of typical developmental changes across the adolescent years. For example, other external factors may have greater impacts on the intake of unhealthy food and drink across adolescence, such as peer influences [30]. Further, it is possible that physiological (e.g., hormonal influences related to pubertal development) and neurobiological (e.g., neurodevelopment, particularly in prefrontal regions involved in executive functions) may impact the association between hedonic hunger and unhealthy intake [31,32,33]. Further, the deceleration of change in association between baseline hedonic hunger and unhealthy food and drink intake was greatest between Wave 1 and Wave 2. Given that this corresponds to the first year of transition into high school, it is possible that novel school-related factors affect unhealthy food and drink intake during this time, such as food availability in school, peer influences, and proximity of school to food outlets. Such factors might potentially contribute to variance in unhealthy diet later in high school, which could obscure the predictive capacity of early adolescent traits, such as hedonic hunger. Additional research is needed to determine if there are other important variables that may moderate the strength of the prospective associations found here.

Importantly, despite being commonly conceptualized as a trait-level factor, there is also evidence that hedonic hunger may change over time among some adolescents [34]. Such changes in hedonic hunger may thus be associated with increases or decreases in unhealthy food and drink intake over time. Little is known about what predicts changes in hedonic hunger in adolescence, yet some evidence suggests that changes in emotional disorder symptoms may be a relevant factor [34]. Further studies in this area, including those examining hypothesized mediators of the association between hedonic hunger and unhealthy food and drink intake as well as examining hedonic hunger as a mediator of the association between other salient predictor variables and unhealthy food and drink intake, are warranted.

Notable strengths of this study include the focus on an important developmental period associated with novel autonomy in regard to food and drink decisions (i.e., adolescence), prospective longitudinal data collection with good retention over time, and the large, racially/ethnically diverse sample of youth. The primary limitation of this study was that the constructs of interest were assessed using only self-report questionnaires. BMI was calculated with self-reported height and weight, which is subject to potential self-report biases. Furthermore, hedonic hunger was not measured across all waves of this study, which precluded examination of bi-directional models. Future research should use more rigorous dietary measures, such as detailed 24-hour recall measures and ecological momentary assessment. Further, hedonic hunger has been shown to fluctuate across the day in adolescents [17], suggesting that the use of ecological momentary assessment to capture variability in hedonic hunger may be useful in future research. This setting of this study was Los Angeles county, and thus, results may not generalize to adolescents in other areas of the United States or globally.

## 5. Conclusions

In conclusion, results suggest that hedonic hunger is an important factor associated with unhealthy patterns of food and drink intake across adolescence. This may have important clinical implications for obesity and eating disorder prevention and health promotion in childhood and adolescence. Assessment of hedonic hunger early in high school could be useful in identifying youth who are at risk for elevated unhealthy food and drink consumption, particularly during the first few years of high school. Efforts to reduce hedonic hunger in youth or improve strategies to cope with/resist the excessive drive to consume typically highly palatable foods may decrease unhealthy food and drink intake behaviors during this developmental period [35]. More research will be needed to determine how best to address hedonic hunger during adolescence, including in health promotion intervention and prevention programs. Additional studies also will be needed to elucidate risk factors for greater hedonic hunger in childhood and adolescence.

## Figures and Tables

**Table 1:** Descriptive statistics and bivariate correlations among study variables.

Variable	1	2	3	4	5	6	7
1. Wave 1 Body mass index	-	−0.08 ***	−0.09 ***	−0.04	−0.02	−0.01	−0.003
2. Wave 1 Highest parental education		-	0.04 *	−0.08 ***	−0.04 *	−0.04 *	−0.05 *
3. Wave 1 Hedonic hunger			-	0.36 ***	0.19 ***	0.18 ***	0.16 ***
4. Wave 1 Unhealthy food and drink intake				-	0.39 ***	0.41 ***	0.38 ***
5. Wave 2 Unhealthy food and drink intake					-	0.41***	0.38 ***
6. Wave 3 Unhealthy food and drink intake						-	0.51 ***
7. Wave 4 Unhealthy food and drink intake							-
*M*	21.68	3.65	2.40	2.58	2.35	2.23	2.19
*SD*	4.33	1.94	0.96	1.03	1.13	0.96	0.94

Note *** *p* < 0.001; * *p* < 0.05

**Table 2:** Multilevel model of baseline hedonic hunger as a predictor of unhealthy food and drink intake over time.

Variable	*B*	*SE*	*p*
Age	−0.05	0.03	0.03
Gender ^a^	−0.12	0.02	<0.001
Race ^b^			
American Indian or Alaskan Native	−0.11	0.10	0.26
Asian	−0.30	0.04	<0.001
Black or African-American	0.22	0.05	<0.001
Hispanic or Latino	0.05	0.03	0.11
Native Hawaiian or Pacific Islander	−0.08	0.05	0.12
Other	−0.06	0.05	0.21
Multiracial	−0.05	0.08	0.56
Missing	0.16	0.08	0.04
Body mass index	−0.006	0.003	0.05
Highest parental education	−0.02	0.01	0.004
Hedonic hunger	0.60	0.05	<0.001
Wave	0.24	0.13	0.05
Wave-squared	−0.04	0.03	0.13
Hedonic hunger × Wave	−0.25	0.05	<0.001
Hedonic hunger × Wave-squared	0.04	0.01	<0.001

Note. ^a^ Reference group is female; ^b^ Reference group is non-Hispanic White.

## Supplementary Materials

The following are available online at https://www.mdpi.com/1660-4601/17/24/9375/s1, Table S1: Multilevel model of baseline hedonic hunger on unhealthy sweet drink intake over time, Table S2: Multilevel model of baseline hedonic hunger on unhealthy sweet food intake over time, Table S3: Multilevel model of baseline hedonic hunger on unhealthy high fat food intake over time.
