# Supplementary material for: Longitudinal Prospective Association between Hedonic Hunger and Unhealthy Food and Drink Intake in Adolescents
Source: Int J Environ Res Public Health. 2020 Dec 15;17(24):9375. doi: 10.3390/ijerph17249375 (PMC7765186; doi:10.3390/ijerph17249375)
Supplement: Supplementary file 1 [file ijerph-17-09375-s001.pdf]

Supplementary Table S1. Multilevel model of baseline hedonic hunger on unhealthy sweet drink intake over time.

Variable	*B*	*SE*	*p*
Age	-0.10	0.03	.01
Gender[a]	-0.33	0.03	<.001
Race[b]			
American Indian or Alaskan Native	-0.10	0.14	.49
Asian	-0.31	0.05	<.001
Black or African-American	0.42	0.07	<.001
Hispanic or Latino	0.19	0.04	<.001
Native Hawaiian or Pacific Islander	-0.27	0.07	<.001
Other	0.02	0.06	.73
Multiracial	-0.03	0.11	.82
Missing	0.27	0.11	.02
Body mass index	0.002	0.004	.59
Highest parental education	-0.04	0.01	<.001
Hedonic hunger	0.61	0.07	<.001
Wave	0.12	0.18	.49
Wave-squared	-0.04	0.04	.32
Hedonic hunger x Wave	-0.27	0.07	<.001
Hedonic hunger x Wave-squared	0.04	0.01	.004

Note. [a]Reference group is female; [b]Reference group is Non-Hispanic White.

Supplementary Table S2. Multilevel model of baseline hedonic hunger on unhealthy sweet food intake over time.

Variable	*B*	*SE*	*p*
Age	-0.08	0.03	.004
Gender[a]	0.11	0.02	<.001
Race[b]			
American Indian or Alaskan Native	0.04	0.12	.74
Asian	-0.15	0.04	.001
Black or African-American	0.13	0.06	.05
Hispanic or Latino	-0.08	0.04	.04
Native Hawaiian or Pacific Islander	-0.02	0.07	.81
Other	-0.15	0.05	.01
Multiracial	0.14	0.10	.17
Missing	0.09	0.10	.37
Body mass index	-0.02	0.003	<.001
Highest parental education	-0.01	0.01	.03
Hedonic hunger	0.64	0.06	<.001
Wave	0.34	0.16	.03
Wave-squared	-0.05	0.03	.08
Hedonic hunger x Wave	-0.27	0.06	<.001
Hedonic hunger x Wave-squared	0.04	0.01	.002

Note. [a]Reference group is female; [b]Reference group is Non-Hispanic White.

Supplementary Table S3. Multilevel model of baseline hedonic hunger on unhealthy high fat food intake over time.

Variable	*B*	*SE*	*p*
Age	-0.02	0.03	.33
Gender[a]	-0.17	0.02	<.001
Race[b]			
American Indian or Alaskan Native	-0.15	0.10	.14
Asian	-0.35	0.04	<.001
Black or African-American	0.29	0.05	<.001
Hispanic or Latino	0.08	0.03	.01
Native Hawaiian or Pacific Islander	-0.03	0.06	.50
Other	-0.02	0.05	.67
Multiracial	-0.18	0.08	.03
Missing	0.17	0.08	.04
Body mass index	-0.002	0.003	.42
Highest parental education	-0.01	0.01	.35
Hedonic hunger	0.59	0.05	<.001
Wave	0.22	0.13	.09
Wave-squared	-0.02	0.03	.21
Hedonic hunger x Wave	-0.24	0.05	<.001
Hedonic hunger x Wave-squared	0.04	0.01	.001

Note. [a]Reference group is female; [b]Reference group is Non-Hispanic White.
